# Atypical antipsychotic agents; Peas in a pod or chalk and cheese?

**DOI:** 10.1186/s12916-014-0126-1

**Published:** 2014-08-01

**Authors:** Ajeet B Singh, Andrew A Nierenberg, Lakshmi N Yatham, Michael Berk

**Affiliations:** IMPACT Strategic Research Centre, School of Medicine, Deakin University, PO Box 281, Geelong, 3220 Australia; Orygen Youth Health Research Centre, Centre for Youth Mental Health, University of Melbourne, 35 Poplar Road, Parkville, Victoria 3052 Australia; Barwon Health and the Geelong Clinic, Swanston Centre, PO Box 281, Geelong, Victoria 3220 Australia; Florey Institute for Neuroscience and Mental Health, Kenneth Myer Building, Royal Parade, Parkville, Australia; Department of Psychiatry, University of Melbourne, 3052 Parkville, Australia; Bipolar Clinic and Research Program, Massachusetts General Hospital, Harvard Medical School, 50 Staniford Street, Boston, MA 02114-2517 USA; Department of Psychiatry, University of British Columbia, Vancouver, BC V6T 1Z3 Canada

**Keywords:** Atypical antipsychotics, Risperidone, Bipolar, Schizophrenia, Generic, Health economics

## Abstract

With escalating health expenditure and a shrinking purse, there is increased focus on the cost efficacy of still patented versus generic medications in general, and for atypical antipsychotics in particular. In a recent *BMC Medicine* article, Godman and colleagues presented data indicating poor uptake of the off patent atypical antipsychotic risperidone, arguing for authorities to mandate its greater use. This is under the assumption of clinical equivalence of atypical antipsychotics. This commentary argues that there are clinically meaningful differences between atypical antipsychotics and important inter-individual heterogeneity in clinical response and tolerability. Access to a broad range of atypical antipsychotics enables clinicians to tailor care, taking consideration of differential efficacy and adverse effects profile in order to meet the needs of individual patients with improved real world effectiveness of treatment. Restriction of agent choice risks detracting from optimal clinical care, with possible poorer outcomes and greater costs of care. A balance between encouraging use of cheapest in class agent and allowing access to various atypical agents for tailored care is likely to produce optimal health outcomes.

Please see related article: http://www.biomedcentral.com/1741-7015/12/98.

## Background

In an economic climate characterised by rising public debt, sluggish economic growth and rapidly expanding health care expenditures, there is increasing pressure on restraining the pace of growth of the health care budget. Pharmaceutical expenditure is a large and growing segment of this budget and an attractive target for cost reduction. In an article recently published in *BMC Medicine*, Godman and colleagues [1] present data from an international retrospective association study of risperidone prescribing rates 20 months before and 20 months after cheaper generic brands became available in several European countries. Interestingly, their data indicate that once generic risperidone was available, it was prescribed less (as a proportion of all atypical antipsychotic scripts), and there was a wide variance between countries in the proportion of risperidone scripts that were generic versus brand name. Furthermore, among newly initiated patients prescribed atypical antipsychotics there was no increased prescribing of generic risperidone when it may have been a valid treatment option. The authors argue that their data have significant implications for health care costs and suggest that health authorities encourage prescribing of cheaper generics versus allowing prescribers to tailor treatment to patient needs based on differential medication side effect and efficacy profiles. We submit that third party payers mandating the use of cheaper generic atypical antipsychotics as first line agents in schizophrenia and bipolar disorders raises important but complex issues meriting debate.

### Differential efficacy profiles of atypical antipsychotics

Atypical antipsychotics are now among the most widely used agents and the bulk of this use - at least in western countries - is for non-psychotic indications, principally mood disorders. The efficacy of individual atypical antipsychotic agents varies by both condition (schizophrenia or bipolar disorder) and phase of illness (particularly bipolar depression). As an exemplar, clozapine has established greater symptom efficacy than other atypical antipsychotics in the treatment of schizophrenia [2], but due to side effect and toxicity profile is not used as a first line agent [3]. Subtle differences in efficacy between other atypical antipsychotics in the treatment of schizophrenia - particularly in improving negative symptoms - arguably exist, but it is unclear whether this is an artefact of methodological variance [2,4]. Importantly, while atypical antipsychotics have equal efficacy in treating mania, they clearly have differential efficacy in the depressive phase of bipolar disorder [5,6], with agents such as quetipine and lurasidone demonstrating efficacy while aripiprazole, ziprasidone and risperidone have failed to show consistent benefit and, hence, are not recommended for the management of the depressive phase of bipolar disorder [6]. Similarly, in unipolar depression, only quetiapine has been shown to be effective in monotherapy. Atypical antipsychotics are also widely used as adjuncts to antidepressants to treat refractory unipolar major depression, and here again, while quetiapine, aripiprazole, risperidone and olanzapine have efficacy based on meta-analytical data, the number needed to treat for response and remission are much higher and the number needed to harm are much lower for olanzapine compared with other atypical antipsychotics [7]. Furthermore, another atypical antipsychotic ziprasidone does not have demonstrated efficacy [8,9]. Differential efficacy of atypical antipsychotics for other clinical uses has been less thoroughly investigated, but risperidone may have utility in dementia associated agitation and obsessive compulsive disorder, but poorer efficacy for generalized anxiety disorder than some other atypical agents, such as quetiapine [10]. Indeed, this inconsistent pattern of efficacy of antipsychotic agents in non-psychotic disorders argues strongly against the presence of a class effect. These agents, in reality, have markedly divergent pharmacodynamics and pharmacokinetics and, while there is consensus that activity against dopamine type 2 receptors is necessary for antipsychotic efficacy [11], there is considerable uncertainty as to which of the multiplicity of actions these agents have may drive their effects in mood disorders [12,13].

### Differential tolerability profiles of atypical antipsychotics

Atypical antipsychotics also have widely differing side effect and tolerability profiles. This is of critical clinical importance as tolerability is one factor driving adherence [14], and medication adherence markedly influences both clinical course and cost of care [15,16]. In the case of risperidone, more extrapyramidal side effects, greater prolactin elevation, and greater weight gain than with some other atypical antipsychotics have been described in recent high profile reviews [2,17]. Elevation of prolactin appears to be particularly marked with risperidone compared to other atypical agents [2] and is associated with hypogonadism, reproductive dysfunction, gynecomastia and bone loss [18]. Osteoporosis and fracture risk is an adverse effect of diverse psychotropic agents attracting increasing recent attention, and one where very clear between-agent differences are apparent [19,20]. Risperidone is, however, less associated with the metabolic syndrome than other agents, particularly olanzapine, quetiapine and clozapine [6]. Trend level differences for all cause medication discontinuation have been noted with risperidone, trending toward greater discontinuation than some other atypical agents – putatively due to differential side effect profiles and tolerability [2,17]. As adverse events are idiosyncratic and unpredictable, the availability of various atypical antipsychotics enables patient and prescriber to tailor treatment based on differential side effects profile, and this may enhance adherence [21]. This clinical need is reflected in international clinical practice guidelines on the management of schizophrenia and bipolar disorder – with several atypical agents considered first line options in patient care [3,6].

## Conclusions

Finite health resources make cost effective use of pharmaceuticals an important societal issue. Atypical antipsychotics have differing tolerability and efficacy profiles. Effectiveness in naturalistic settings is highly dependent on subjective efficacy as well as on adherence, which in turn is related to long and short term side effect profiles. While access to cheaper generic atypical antipsychotics offers an opportunity for more cost effective care, it is not without risks. Mandating switching to a generic atypical antipsychotic without any corresponding clinical indication may result in increased risk of relapse, reduced adherence, poorer outcomes and greater ultimate health care costs [22,23]. Reducing access to a range of atypical agents as first line treatment will hamper tailoring of medication to individual patient needs and preference, reducing clinical effectiveness and making it more difficult for clinicians to follow current best practice guidelines [3,6]. Nevertheless, there will be some clinical instances where use of a more cost-effective generic atypical agent (either first line or as switch to agent) may be appropriate, and ways for third party payers to encourage such behaviour merit further exploration. In the absence of data, we urge policy makers to strike a balance between tailored effective care (with choice of atypical antipsychotic agent) versus mandated use of cheapest in class agent. To obtain the data necessary for evidence-based policy, it would be useful to invest in comparative effectiveness studies that focus on: 1) outcomes of tailored versus mandated care, and 2) sufficiently powered cluster-randomized studies of key atypical antipsychotics. Our patients’ health deserves no less.
